# Comparison of Head and Neck Cancer Distribution in Turkish and Syrian Populations

**Published:** 2019-10

**Authors:** Gozde SERİNDERE, Behiye BOLGUL, Didar GURSOY, Sibel HAKVERDİ, Nazan SAVAS

**Affiliations:** 1. Department of Dentomaxillofacial Radiology, Faculty of Dentistry, Hatay Mustafa Kemal University, Hatay, Turkey; 2. Department of Pedodontics, Faculty of Dentistry, Hatay Mustafa Kemal University, Hatay, Turkey; 3. Department of Pathology, Faculty of Medicine, Hatay Mustafa Kemal University, Hatay, Turkey; 4. Department of Public Health, Faculty of Medicine, Hatay Mustafa Kemal University, Hatay, Turkey

**Keywords:** Head and neck, Cancer, Pathology, Epidemiology

## Abstract

**Background:**

Although oral health improves in several countries, global problems are still present. Predictably, the disadvantaged and poor population groups in both developing and developed countries have high rate of malign disease. The aim of this study was to evaluate the prevalence of head and neck cancers (HNCs) and to compare them between Syrian and Turkish population.

**Methods:**

A total of 4570 patients confirmed to have HNC histopathologically from Hatay Mustafa Kemal University Hospital Pathology report archive were retrospectively evaluated. Among them, 452 were Syrian patients while 4118 were Turkish patients. Data were collected from 2010 to 2017. Gender and age information were taken from medical records. According to the pathological results, HNCs were classified.

**Results:**

In 474 patients, HNCs were inscriptived, of which 317 were in males and 157 in females aged 23–80 years with histologically approved cancer of head and neck area. Overall, 100 were Syrian patients while 374 were Turkish patients. In both Syrian and Turkish patients, the most observed HNC was squamous cell carcinoma (SCC).

**Conclusion:**

Nowadays, the prevalence of cancer is higher because of the excessive consumption of alcohol, tobacco, chewing, and smoking. For the higher cancer incidence in Syrian refugees, we thought that the impact of war such as stress may have been effective as well as the known several etiologic factors of cancer. For the increased risk of cancer, the early diagnosis of this become more important.

## Introduction

Head and neck cancers (HNCs) are termed as malignant lesions which derives from nasal cavity, paranasal sinuses, oral cavity, pharynx, and larynx. The greater part of head and neck malignancies (90%) constitutes from squamous cell carcinoma (SCC) which the usage of tobacco and alcohol is the most remarkable risk factors (1).

HNCs are the sixth most frequent type of cancer which involves approximately 6% of all cases and about 650,000 new cases are diagnosed in every year, additionally 350,000 deaths are annually present in worldwide. The average age of diagnosis is in the sixth decade of life and the male-to-female predominance is substantially present (2).

Although incidence of HNCs has a slight decrease in the past 2 decades (3), the region of tongue base and tonsil has an increase with regards to cancer (4).

In most studies that research the direct healthcare costs of HNCs, it was benefited from United States database for public and private payers. These studies reported that costs are usually higher for HNC patients who had recurrent and/or metastatic disease, surgical operations and for patients insured by private payers. Further studies will be useful especially in Europe and other regions outside the United States of America. So, prospective studies about the cost of patients with HNCs, can provide more systematic comparison of costs, and important economic information to patients, payers and providers (5). In 2012, the International Agency for Research on Cancer reported the role of human papillomavirus type 16 for occurrence of or pharyngeal cancer (6). A strong association was determined between Epstein-Barr virus and nasopharyngeal cancer. The eating of cured meats are also defined as a risk factor in addition with smoking (7). This article gives information about the prevalence and distribution of HNCs according to localization and histopathologic results among Turkish and Syrian patients. Additionally, the cause, symptoms, diagnosis and management of HNCs is discussed.

## Materıals and Methods

Between the years of 2010 and 2017, a total of 452 Syrian patients and 4118 Turkish patients referred to Department of Pathology, Department of Dentomaxillofacial Radiology, Faculty of Medicine, Faculty of Dentistry, Hatay Mustafa Kemal University, Turkey were retrospectively evaluated.

Informed consent from the patients was not needed for this study. All cases which were histopathologically diagnosed as HNC constitutes to the study.

The inclusion criteria:

- The patients who are Syrian and Turkish
- Only the HNC cases diagnosing with the histopathological assessment.

The exclusion criteria:

- Patients with the other nationalities
- The cases that were not confirmed by histopathological analysis.

The cases were recorded according to gender, age and location of the cancer. The all HNC cases were also classified according to the histopathological diagnosis.

SPSS 21 (Chicago, IL, USA) was used for statistical analysis. It was benefited from Chi-Square tests, Fisher's exact (1-sided) test and Independent Samples test for assessment the difference between the means of two independent or unrelated groups. Differences were supposed significant at *P*<0.05.

## Results

**Fig. 1a, b:**
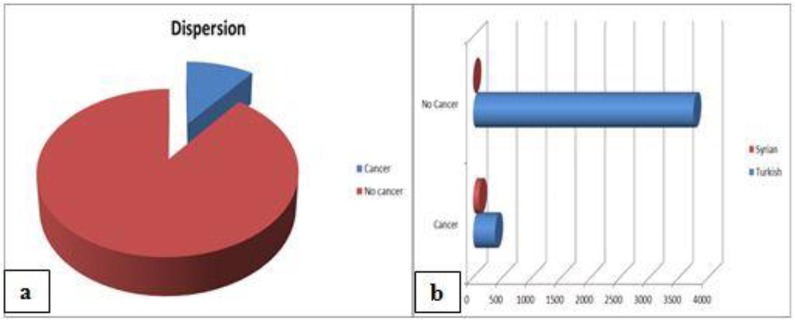
Distribution of the incidence of cancer in the head and neck region

From 2010 to 2017 we evaluated 4570 head and neck lesions (4118 in Turkish patients, age mean 41.3 yr SD ±21.2; 452 in Syrian patients, age mean 37.3 SD ±22.8 yr)confirmed by histopathological analysis (Fig. 1a, b). The mean age of Turkish and Syrian males was 41.4 and 41.1 yr, respectively. However, the mean age of Turkish and Syrian females was 41.06 and 30.3 yr, respectively.

In 474 patients (317 males; 157 females), HNCs were observed with a range from 5 to 94 years. Among these patients, 100 (69 males; 31 females) were Syrian patients while 374 (248 males; 126 females) were Turkish patients (Table 1).

**Table 1:** Dispersion of HNCs cancer according to the population

***Population***	***Patients with cancer N (%)***	***No Cancer N (%)***	***Total N (%)***
Turkish	374 ( 9.1)	3744 (90.9)	4118 (100)
Syrian	100 (22.1)	352 (77.9)	452 (100)
Total	474 (10.4)	4096 (89.6)	4570 (100)

**Fig. 2:**
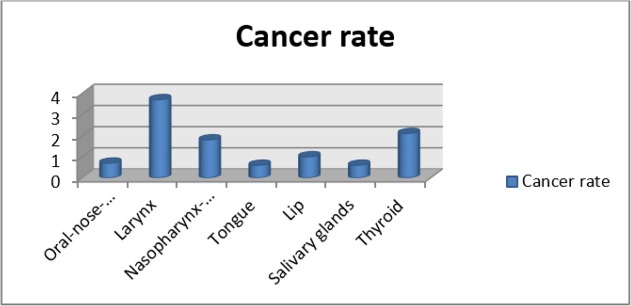
Distribution of head and neck cancer according to the location

Incidence of HNCs increased by age. Considering the gender in all the age groups, males were more affected than females. In both Syrian and Turkish patients, the most observed cancer location was larynx and the most observed cancer type was SCC (Fig.2, Table 2).

**Table 2:** Dispersion of the incidence of cancer in the head and neck according to the gender and site

***Region***	***Male***	***Female***	***Turkish***	***Syrian***	***No cancer***	***Cancer rate (%) (According to total patient number)***
Oral-nose-nasal cavity	25	8	22	11	547	0.7
Larynx	157	11	128	40	406	3.7
Nasopharynx-Tonsil	42	41	68	15	1391	1.8
Tongue	18	8	18	8	197	0.6
Lip	34	10	33	11	281	1.0
Salivary glands	13	13	22	4	329	0.6
Thyroid	28	66	83	11	945	2.1
Total	317	157	374	100	4096	-

According to the pathological types, in Syrian patients, except salivary glands and thyroid, SCC was most frequently observed. In Turkish patients, except nasopharynx-tonsil and thyroid, SCC was the most common cancer in other regions (Table 3, 4). Statistically significant differences were found between nationality and cancer region indicated thyroid, lip, tongue, oral, nose mucosa and nasal cavity (*P*<0.05) while no statistically significant differences were found between nationality and cancer of larynx, salivary glands, nasopharynx and tonsil.

**Table 3:** Dispersion of HNCs according to the region in Syrian patients. Percentages are shown in parenthesis. Totals may not equal 100 because of indicating most and least common cancers. Sq. Cell Ca, squamous cell carcinoma, Bas. Cell Ca, Basaloid cell carcinoma, Adeno Ca, Adenocarcinoma

***Region***	***Most Common***	***Least Common***
Oral-nose-nasal cavity	Sq. Cell Ca. (27.2) Bas. Cell Ca. (18.1) Mucoepidermoid (18.1)	Non-Hodgkin Lymphoma (9.09) Malignant melanoma (9.09)
Larynx	Sq. Cell Ca. (100)	-
Nasopharynx-Tonsil	Sq. Cell Ca. (40) Nasopharygeal ca (26.6)	Hodgkin (6.6) Malignant mesenchymal tumour (6.6)
Tongue	Sq. Cell Ca. (87.5)	Borderline (12.5)
Lip	Sq. Cell Ca. (80)	Bas. Cell Ca. (10) Sq. Cell Ca.+ Bas. Cell Ca. (10)
Salivary glands	Adeno Ca (50)	Sq. Cell Ca. (25) Basaloid SCC (25)
Thyroid	Papillary Ca (90.9)	Medullary Ca (9.1)

**Table 4:** Dispersion of HNCs according to the region in Turkish patients. Percentages are shown in parenthesis. Totals may not equal 100 because of indicating most and least common cancers. Sq. Cell Ca, squamous cell carcinoma, Adenoid cystic, Adenoid cystic carcinoma, Mucoepidermoid, Mucoepidermoid carcinoma

***Region***	***Most Common***	***Least Common***
Oral-nose-nasal cavity	Sq. Cell Ca. (71.4)	Bas. Cell Ca. (4.7) Mucoepidermoid (4.7)
Larynx	Sq. Cell Ca. (100)	-
Nasopharynx-Tonsil	Non-Hodgkin (44.1) Sq. Cell Ca. (17.6) Nasopharyngeal Ca (22.1)	Mucoepidermoid (1.47) Malignant melanoma (1.47)
Tongue	Sq. Cell Ca. (100)	-
Lip	Sq. Cell Ca. (93.75)	Bas. Cell Ca. (6.25)
Salivary glands	Sq. Cell Ca. (27.2) Mucoepidermoid (18.1) Malign epithelial tumour (18.1) Non-Hodgkin (13.6)	Malignant melanoma (4.5) Adenoid cystic (4.5)
Thyroid	Papillaryca (93.9)	Medullary ca (6.1)

According to Levene's test for equality of variances, sample variances were not homogeneous. Statistically significant differences were found between the mean age of Turkish and Syrian population. No statistically considerable differences were found between the mean age of males while statistically considerable differences were found between the mean age of females.

## Discussion

Head and neck cancer happens in several tissue types and sites, requiring multidisciplinary approach. Although it is less common observed with incidence as 270 cases/million than some cancers such as lung (620 cases/ million) and colon cancer (550 cases/million), the numbers do not completely reflect the effect of the illness. Patients suffering from HNC characteristically live longer than the patients with lung cancer and probably have more morbidity than the patients with colon cancer (8–10).

According to some studies (11–13), HNCs, especially oral cancer, is increasing in young individuals in the world. Despite these studies, in our study, the mean age was approximately mid-forties.

The rate of oral cancer decreased in males and there was no change in females except in the age group 40–59 with a significant decrease (14). In another study (15), about the prevalence of oral cancer and oral SCC in Mexicans, an increase the frequency of oral cavity was reported. This increment was reported in both genders but only in females it was significant. In our study, there was male predominance in both Syrian and Turkish patients with oral cancer.

Davies and Welch (16) achieved community-based data for each of the 13 anatomic sites from the SEER program and evaluated approximately 75,000 new reports of HNCs in 2001. The most common of 5 areas were thyroid (29%), larynx (15%), oropharyngeal mucosa (12%), tongue (10%) and soft tissue (9%). Cancers of the other areas were less often. In many areas, SCC was more commonly reported. Similarly, in our study, larynx and thyroid was found as the most common regions for HNCs and the most frequently found cancer type was SCC.

Blomberg et al (17) studied about the overall trends in the incidence of HNC and the effect of human papillomavirus on cancer entity and frequency in Danish individuals in 1978–2007 with data obtained from the nationwide Cancer Registry database. Among 26,474 cases, SCC was the major cancer type (88.6%), 4.2% were adenocarcinomas and 1.2% were mucoepidermoid carcinomas (principally in the salivary glands for both of them), 2.9% were unspecified epithelial carcinomas. The three most common areas were reported as oral cavity (27.8%), larynx (27.7%) and lip (12.4%). In our study, for Syrian patients, the most common regions were larynx and nasopharynx-tonsil, respectively. In Turkish patients, similarly to the study of Davies and Welch (16), the most common regions of cancer were larynx and thyroid, respectively. Similar to the study of Blomberg et al (17), the most common observed cancer type was SCC and thyroid papillary cancer. The median age for diagnosis is in early sixth decade with a male predominance (2). In our study, male predominance was similarly found in both populations except thyroid cancer. The mean age was 37.3 in Syrian patients while 41.3 in Turkish patients. In our hospital, diagnosis of HNCs was performed in earlier age. This was an advantage for the health of the patients.

Symptoms depend on the region of the primary cancer, these can be difficulty in the act of swallowing, hemorrhage or blockage of nasal area, hoarseness, ear pain, enlargement of cervical lymph nodes and/or nonhealing wounds or ulcers in oral mucosa (18).

It is important to determine certain staging of cancer for the decision of the medical treatment. There is some staging methods such as investigation by a head and neck surgeon and radiological evaluation with computed tomography or magnetic resonance imaging, or both of them (19).Using of positron emission tomography scanning with [18F] fluoro-2-deoxyglucose increases, especially to determine nodal or distant metastasis, or both of them may be analyzed (20).Combination of positron emission tomography and computed tomography scans provides more certain information to describe HNCs (21). Especially for thyroid cancer follow up, neck ultrasonography is the most sensitive modality for metastatic lymph nodes diagnosis in patients with thyroid cancer (22).

In general, 60%–65% of patients suffering from HNC may be treated by way of surgery and/or radiotherapy. The single treatment such as either surgery or radiotherapy are performed in patients who have early-stage (I and II) disease but in patients who have stages III and IV tumor, the treatment requires a multi-approach analysis such as comprehensive surgery and radiotherapy or chemo radiation. Besides, a smaller fraction (30%) can be treated. The reasons of treatment unsuccessful include loco regional recurrences (60%), metastatic disease (up to 30%) and second primaries, respectively (23). The prognosis for patients is based on the area, grade and extension of tumor, presence of nodal involvement (24).

HNC patients who survive, frequently needcomprehensivetreatment such as speech therapy, swallowing and dentomaxillofacial rehabilitation, also physical and vocational therapies (25). The physical and mental work talent may be related to the result of the employment status of cancer survivors. As a result of the advanced and new cancer treatments with vocational therapies, cancer survivors can expect better life quality after surviving cancer (26).

The dentists, the radiologists and pathologists have an important role to assess HNCs. From day to day, the prevalence of HNC increases. Bukhari et al. (27) reported that neck mass was the most frequently observed in head and neck subspecialty of Ear Nose Throat in patients who presented to the emergency department. Additionally, according to the study of Chida et al. (28), stress-related psychosocial factors cause negative effect on cancer development. In our study, the observed high cancer rates among Syrian patients may be the result of stress, socioeconomic conditions and nutritional disorders.

A possible limitation of this study is that the prevalence belongs to all new patients and the patients who previously referred, and that not provide the possibility of developing a disease in healthy individuals in a period of time.

## Conclusion

To our knowledge, this is the first study evaluating the incidence of HNCs with pathological types in Turkish and Syrian population. Additionally, it is not possible to understand whether or not the disease occurs in the continuation of etiologic factors. Further studies are needed to confirm the incidence of HNCs and the reason of higher cancer rates in Syrian patients.

## Ethical considerations

Ethical issues (Including plagiarism, informed consent, misconduct, data fabrication and/or falsification, double publication and/or submission, redundancy, etc.) have been completely observed by the authors.
